# Details of development of the resource for adults with asthma in the RAISIN (randomized trial of an asthma internet self-management intervention) study

**DOI:** 10.1186/s12911-015-0177-z

**Published:** 2015-07-28

**Authors:** Deborah Morrison, Frances S. Mair, Rekha Chaudhuri, Marilyn McGee-Lennon, Mike Thomas, Neil C. Thomson, Lucy Yardley, Sally Wyke

**Affiliations:** General Practice & Primary Care, Institute of Health & Wellbeing, University of Glasgow, 1 Horselethill Road, Glasgow, G12 9LX UK; Institute of Infection, Immunity and Inflammation, Gartnavel General Hospital, University of Glasgow, 1053 Great Western Road, Glasgow, G12 0YN UK; Computer and Information Science, University of Strathclyde, Glasgow, G1 1XH UK; Primary Care Research, Aldermoor Health Centre, University of Southampton, Aldermoor Close, Southampton, SO16 5ST UK; Department of Psychology, University of Southampton, Shackleton Building, Highfield, Southampton, SO17 1BJ UK; Institute of Health and Wellbeing, College of Social Sciences, University of Glasgow, Rm 204, 25-28 Bute Gardens, Glasgow, G12 8RS UK

**Keywords:** Asthma, Self-management, Adherence, E-health, Randomized controlled trial, Complex intervention, Inhaled corticosteroids, Internet, Behaviour change, Lifeguide

## Abstract

**Background:**

Around 300 million people worldwide have asthma and prevalence is increasing. Self-management can be effective in improving a range of outcomes and is cost effective, but is underutilised as a treatment strategy. Supporting optimum self-management using digital technology shows promise, but how best to do this is not clear. We aimed to develop an evidence based, theory informed, online resource to support self-management in adults with asthma, called ‘Living well with Asthma’, as part of the RAISIN (Randomized Trial of an Asthma Internet Self-Management Intervention) study.

**Methods:**

We developed Living well with Asthma in two phases.

Phase 1: A low fidelity prototype (paper-based) version of the website was developed iteratively through input from a multidisciplinary expert panel, empirical evidence from the literature, and potential end users via focus groups (adults with asthma and practice nurses). Implementation and behaviour change theories informed this process.

Phase 2: The paper-based designs were converted to a website through an iterative user centred process. Adults with asthma (*n* = 10) took part in think aloud studies, discussing the paper based version, then the web-based version. Participants considered contents, layout, and navigation. Development was agile using feedback from the think aloud sessions immediately to inform design and subsequent think aloud sessions. Think aloud transcripts were also thematically analysed, further informing resource development.

**Results:**

The website asked users to aim to be symptom free. Key behaviours targeted to achieve this include: optimising medication use (including inhaler technique); attending primary care asthma reviews; using asthma action plans; increasing physical activity levels; and stopping smoking. The website had 11 sections, plus email reminders, which promoted these behaviours. Feedback on the contents of the resource was mainly positive with most changes focussing on clarification of language, order of pages and usability issues mainly relating to navigation difficulties.

**Conclusions:**

Our multifaceted approach to online intervention development underpinned by theory, using evidence from the literature, co-designed with end users and a multidisciplinary panel has resulted in a resource which end users find relevant to their needs and easy to use. Living well with Asthma is undergoing evaluation within a randomized controlled trial.

**Electronic supplementary material:**

The online version of this article (doi:10.1186/s12911-015-0177-z) contains supplementary material, which is available to authorized users.

## Background

Asthma is common, affecting 300 million people worldwide, and its prevalence is increasing; an estimated additional 100 million people will have asthma by 2025 [16]. Despite increasing costs associated with asthma [2], care remains suboptimal, with many patients tolerating symptoms and lifestyle limitations unnecessarily, due to suboptimal use of proven available therapies [11, 26, 29, 32].

Supporting optimum self-management by providing relevant self-management education including how to use an asthma action plan (AAP), regular health professional review, and optimal use of medications has been shown to have positive effects on a range of asthma outcomes such as improved quality of life, lower rates of healthcare contacts, and fewer days off work and school [7]. Promoting self-management is a recommendation in worldwide asthma guidelines [4, 8]. Despite this, self-management as a treatment strategy remains underused [8, 32, 34]. Recently, new information and communication technologies (ICTs) have been proposed as a means to improve asthma self-management uptake. We conducted a meta-review [22] which showed that online interactive resources to support self-management of asthma can be safe and effective at improving some outcomes such as markers of self-care, activity limitation, quality of life and medication use. However, interventions were poorly described and it was impossible to extract generalisable lessons about the key ‘active ingredients’ of interventions. This challenge has previously been recognised by Michie et al. [20] who in response have developed a taxonomy of behaviour change techniques (BCTs) which they propose researchers can use to describe interventions, in order to overcome the lack of a systematic way of determining the ‘active ingredients’ of a complex interventions [21].

Traditionally, development of online interventions has been resource intensive with each intervention requiring to be programmed individually by a team of programmers from scratch—a barrier to internet based interventions being cost-effective when compared to face-to-face or paper alternatives. A team in Southampton who have recognised the increasing potential for delivering health care online have developed an open access software package called LifeGuide [10, 38, 41]. LifeGuide aims to allow researchers from a non-computer programming background to more easily and flexibly create and modify internet-delivered interventions. It has been used successfully in a number of health related interventions [3, 33, 38]. A key design feature of LifeGuide is that it allows researchers to easily test parts of an intervention and immediately modify and improve it based on the findings, and to trial it in the development phase.

Here we describe the development and optimisation of the Living well with Asthma website which we developed using the LifeGuide open access software package guided by the updated MRC guidance for the development and evaluation of complex interventions [18]. The Living well with Asthma website is currently undergoing evaluation in a randomised controlled trial (RCT) called RAISIN (Randomized Trial of an Asthma Internet Self-Management Intervention) (ISRCTN 78556552) [23].

## Methods

To develop the Living Well with Asthma website we followed the steps outlined in the updated MRC guidance which recommends that intervention development should be systematic, include review of the evidence, be theory based, and incorporate feasibility or user testing [18]. In this section we describe the two phases of work we undertook to incorporate these steps. Ethical approval was granted from the West of Scotland Research Ethics Committee (12/WS/0068), and all participants provided informed consent.

### Phase 1: intervention planning

Phase 1 describes the process of developing a ‘first draft’ of the website. This phase consisted of three main work packages (WP), all overseen by a multidisciplinary ‘expert panel’ made up of 3 general practitioners, a respiratory physician, a health psychologist, a social scientist, and a human-computer-interaction researcher.

### Work package 1—understanding the evidence & incorporating theory (scoping review and expert panel)

Campbell et al. [5] describe 5 key tasks involved in defining and understanding the ‘problem’ that your intervention is aiming to solve, namely: 1) defining and quantifying the problem; 2) identifying who is mostly likely to benefit; 3) understanding the pathways which contribute to the problem; 4) consideration of whether (and how) these pathways are amenable to change; 5) and attempting to quantify the potential for improvement. We did a brief scoping review of the literature and used the experience of our expert panel to work through these tasks. We identified a list of features that a resource should have, incorporating recommended behaviour change concepts [25].

### Work package 2—getting user perspectives on a web resource (focus groups)

**Fig. 1:**
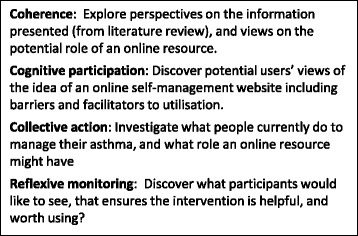
Focus group topic guide (NPT informed)

In order to investigate the plausibility of this list with potential end users we convened 2 focus groups, consisting in total of 9 adults with asthma (6 female, 3 male), and 4 practice nurses who undertake asthma reviews. Recruitment was undertaken using a range of sources: primary care, Asthma UK Research and Policy volunteers, Chest Heart Stroke Scotland volunteers and a secondary care asthma clinic. Adults aged 18 and over were eligible provided they had a diagnosis of asthma and could provide informed consent. There was no upper age limit on participation. Participants could agree to participate in a focus group, up to two think aloud studies (described in phase 2), or both. Focus groups were held at the Department of General Practice & Primary Care, University of Glasgow, and were audio recorded and transcribed. We used the implementation theory Normalization Process Theory (NPT) [24] to inform the topic guide for these focus groups (Fig. 1). This theory was used as it is being increasingly advocated as a means to understand implementation processes and enhance the implementability of interventions [17, 24]. Discussion focused on the perceived barriers and facilitators to sustained use of an online resource to support self-management. This consideration of implementation issues at such as early stage is a key message from the MRC guidance. Our list of potential features derived from WP 1 was explored using questions generated from our topic guide (Fig. 1) and we sought suggestions for additional features. The focus groups were transcribed and any statements which were barriers or facilitators to self-management were extracted, along with any suggestions for features to include in our website.

### Work package 3—developing a draft version of the website (expert panel)

Using information gathered from WP 1 and 2, the list of suggested features to include in the website was reviewed and refined iteratively. As low fidelity prototype pages were generated (initially using Microsoft Word or PowerPoint) (also referred to as draft pages) they were reviewed initially by those in the panel with a clinical background to ensure the content was factually correct. Subsequently the pages were shown to members of the expert panel with specific expertise in behaviour change theory to ensure maximum opportunity for promoting behaviour change was incorporated into each page or section, using the behaviour change concepts agreed on from WP1 [25]. From this a draft version of each potential webpage was finalised, ready for think aloud study evaluation.

### Phase 2: iterative refinement of the resource contents of the website (think aloud studies and expert panel)

Draft pages developed at the end of Phase 1 were gradually translated into interactive webpages with input from potential end users in the form of think aloud studies, and review by the expert panel. While LifeGuide can be used by researchers with no computing science background, due to time constraints, a programmer transferred the majority of the draft pages into LifeGuide initially. Think aloud studies were undertaken by the first author at either the participant’s home, or the Department of General Practice & Primary Care, University of Glasgow, depending on individual participant preference. Participants were recruited from the same pool as the focus groups, and they could participate in a maximum of two think aloud studies. There were two waves of think aloud studies: the first 4 used draft webpages still on paper or PowerPoint slide, the latter 6 were undertaken completely using the prototype webpages on LifeGuide. Participants were asked to say whatever they thought or felt about what they were seeing, with prompts and questions used to elaborate on responses. The participants were then encouraged to voice any additional suggestions or opinions to improve the resource, for example what they liked and disliked, what was intuitive and what was not, and how they envisaged using such a website in real life in the future. The majority of the findings from the think aloud studies were acted upon immediately after the session by the researcher doing the think aloud studies, in order to progress the resource ready for the subsequent think aloud study. We also thematically analysed the transcriptions of the think aloud studies with the aim of providing information for further development of the resource following the pilot RCT. Thematic analysis was undertaken using a coding frame developed by DM. Both DM and SW independently coded the first 2 transcripts, and results compared, after which DM coded the remaining transcripts. Comments were noted to be either a positive comment, where the user liked or identified with what they saw, or a negative comment where the user disliked or disagreed with what they saw, or where the user suggested an improvement or alternative way of presenting the data. The final version of the Living well with Asthma website was formally mapped to Michie and colleagues latest BCT taxonomy [21] in order to describe which BCTs were present. Every page of the website was reviewed by the first author (DM), and where relevant a BCT was assigned. These were subsequently reviewed by SW. We did this to provide a reliable record of the content of this behaviour change intervention, and to confirm that we included a range of BCTs as planned.

## Results

This results section describes the key steps in our website development.

### Phase 1: initial planning stages

#### Work package 1—understanding the evidence & incorporating theory (scoping review and expert panel)

The planning stage focussed on the 5 key tasks outlined by Campbell et al. [5]. This process helped us understand that the main problems we aimed to address centred round: 1) the suboptimal use of preventative therapies; 2) the high levels of symptom burden; and 3) the low rates of attendance at asthma reviews and use of asthma action plans. We anticipated that concepts derived from behaviour change theory should help us address these identified problems, and the full results of WP 1 are described in Table 1.

**Table 1:** Defining and understanding the problem

Key Tasks [5]	Commentary relating tasks to LWWA
Task 1: Define and quantify the problem	Optimum self-management of asthma is an underused, yet proven treatment strategy that improves a range of asthma outcomes (fewer visits to emergency room, hospitalisations, unscheduled visits to doctors, and days off work and school, reduces nocturnal asthma and improves quality of life) [7]. People with asthma have:
	1) Suboptimal use of preventative therapies. Adherence to therapies in long term conditions is around 50 % [39]. Low use of preventative (inhaled corticosteroids (ICS)) therapies and high use of short acting beta agonists (SABA) reliever inhalers, is a pattern commonly seen which is associated with poorer asthma control [29].
	2) High levels of symptom burden (46 % daytime symptoms and 30 % nocturnal symptoms) [30], with lack of recognition of scope for improvement: 50 % of patients reporting severe persistent symptoms report their own asthma as being completely or well controlled [30]. This results in people with uncontrolled or deteriorating asthma not seeking timely medical advice.
	3) Suboptimal attendance at asthma reviews with low use of asthma action plans (AAPs) [13, 32] as evidenced by the National Review of Asthma Deaths (NRAD) where only 23 % of those who died having been provided with an AAP [32], and attendance at asthma reviews in Scotland was only 65 %.
Task 2: Identify and quantify the population most affected, most at risk, or most likely to benefit from the intervention	The Global Initiative for Asthma (GINA) guidelines lists risk factors for poor asthma outcomes [8]:
	• Uncontrolled asthma symptoms
	• Increased use of short acting beta agonist (SABA) e.g. reliever therapy
	• Inadequate inhaled corticosteroids (ICS), including poor technique.
	• Low FEV_1_ (especially if <60 % predicted)
	• Major psychological or socioeconomic problems
	• Smoking
	• Comorbidities: obesity, rhino-sinusitis, food allergy
	• Previous exacerbations or intensive care admissions for asthma
	The majority of these factors are related to uncontrolled asthma symptoms, and therefore a key way of identifying those most likely to benefit is to target those with uncontrolled asthma symptoms.
Task 3: Understand the pathways by which the problem is caused	With reference to problems outlined in task 1:
	1) Reasons for low adherence to asthma therapies are often related to concerns about side effects, or perceptions that they don’t need to be on treatments [12].
	2) The global asthma insights and reality surveys [29] provides evidence of suboptimal asthma control and suggests reasons for it. First, people with asthma overestimate how controlled their asthma is, therefore don’t consider themselves to be candidates for gaining improvement with asthma treatments. Second, those who do acknowledge they have symptoms and limitation of activities accept them as unavoidable consequences of having asthma.
	3) Patients reasons for not attending asthma reviews revolve around feelings that their asthma is not serious enough [9]. Asthma Action plans are underused for several reasons [31]:
	i) Differences in beliefs and attitudes between health care professionals and people with asthma.
	ii) Perceived irrelevance of AAPs of the part of those who would potentially benefit from them
	iii) Health professionals only offer AAPs to select groups of patients (e.g. with well controlled asthma, or those with higher levels of educational achievement).
	In summary, people with asthma often underestimate their symptoms and overestimate their control, not making use of available therapeutic options (medications, AAPs and advice from health professionals). Those who do recognise they have symptoms may not adhere to prescribed medications due to misunderstandings around medication side effects, or perceived benefits of using AAPs.
Task 4: Explore whether these pathways may be amenable to change and, if so, at which points	With specific reference to the three ‘problems’ outlined in Task 1:
	1) Prompting users to consider reasons why they don’t take medications regularly (barriers) and consider strategies to overcome these barriers. Providing information about benefits of inhaled corticosteroids, challenging misconceptions and negative beliefs. Focussing on benefits meaningful to individuals such as fewer days off work, managing that exercise class etc. Providing instructions (ideally including videos) to demonstrate correct inhaler technique.
	2) Promoting the message that users should be aiming for no symptoms. Providing information to challenge the belief that having asthma symptoms is normal, and asking validated questions to determine if users are currently putting up with symptoms, providing feedback on response. Prompting users to recognise if they avoid activities due to their asthma, or are limited in everyday tasks such as housework, gardening, visiting friends. Turn these limitations into ‘goals’ to aim towards, and describing how these goals are achievable for them.
	3) Provide information that people who use AAPs and attend for reviews have fewer symptoms and fewer asthma attacks. Provide quotes from practice nurses encouraging attendance for reviews. Remove physical barrier to using AAPs by providing a template that can be taken to health professionals (identical to those provided by local health board).
	The expert panel will ensure that behaviour change theory is incorporated into the web page contents and full analysis of behaviour change techniques will be done on final website (Table 6).
Task 5: Quantify the potential for improvement	An estimated 300 million people worldwide have asthma and its prevalence appears to be increasing with an estimated additional 100 million people with asthma by 2025 [16]. Depending on criteria used to define poor control evidence suggests that levels of uncontrolled asthma range from at least 25 %, and probably higher [29, 30, 36]. Our primary outcomes in a full scale RCT would be symptom level using a questionnaire. A good candidate would be the Asthma Control Questionnaire and we would aim for a drop of 0.5 in score which is the minimally important clinical difference [14]

### Work package 2—getting user perspectives on a web resource (focus groups)

We shared our results from Table 1 with potential end users in the focus groups. Excluding the practice nurses the average age of participants was 41 years (range 23 to 56). Six participants were female, 4 male, and included participants from highest and lowest deprivation deciles (median 4, IQR 1, 8). Table 2 describes the participants, illustrating which focus group (or think aloud study) they participated in. Participants were recruited from Asthma UK volunteers (*n* = 5), primary care (*n* = 3) and hospital asthma clinic (*n* = 2). Barriers to optimum self-management identified by focus groups included not accepting diagnosis, difficulties keeping track of medications and remembering to order more, and the length of time between asthma reviews resulting in knowledge loss. Facilitators to using an online resource included staggering of information, a resource to bridge the gap between annual reviews and reinforcement of material covered, provision of email reminders i.e. ordering medication and flu vaccinations, resource being promoted during annual reviews and making users aware of different types of inhalers available and importance of finding one that suits.

**Table 2:** Demographics of participants in focus groups and think aloud studies

Participant number^a^	FG 1	FG 2	TA 1^b^	TA 2^b^	Female	Male	Age (yrs)	SIMD^c^	Ethnicity
1		●	● (2)		●		44	1	White British
2	●		●(3)		●		23	1	White British
3		●	● (4)	● (11)	●		51	8	White British
4	●		● (5)	● (9)		●	46	4	White British
5		●	● (6)			●	23	1	White British
6		●	● (7)		●		56	8	White British
7	●		● (8)		●		55	3	White British
8	●					●	41	6	White British
9	●				●		29	10	White British
10			●(1)	● (10)		●	48	10	White British

^a^refers to adults with asthma participating. Two practice nurses also present in each focus group, details not provided. ^b^number in brackets refers to think aloud study number, participant number 3, 4 and 10 participated in two think aloud studies each. ^c^Scottish Index of Multiple Deprivation. Range from 1 (most deprived) to 10 (most affluent)

These barriers and facilitators were combined with those from the literature (including asthma guidelines) to provide a list of suggested features to include in a resource. This process is shown in full Additional file 1, illustrating the rationale for the contents of the website.

### Work package 3—developing a draft version of the website (expert panel)

By the end of WP 1 and 2 we established there were 6 main behaviours we wanted to promote within the website:

- Recognise symptoms, don’t put up with them (aim for no symptoms)
- Optimise medication use (including inhaler technique)
- Attend for regular asthma review
- Use asthma action plans
- Increase physical activity
- Stop smoking

The expert panel reviewed the list of suggested features from Additional file 1 which led to the removal of four: a diary for tracking medication use, a diary for tracking peak expiratory flow (PEF) rate, a tailored action plan and a dedicated family & friends sections. The expert panel felt that evidence and personal experience suggested that use of diary tools was rarely sustained except by a few very motivated individuals. Instead regular prompts to think about current asthma symptoms based on the ‘Royal College Physicians 3 Questions’ (RCP 3Q) screening tool [27] was incorporated throughout the resource and in the automated emails. This asks the user about difficulty sleeping because of asthma, asthma symptoms during the day, and interference with usual activities. If users answer yes to even one question then further assessment of asthma control is indicated [28, 35]. Action plans work best when personalised to the individual [6] and the IT requirements of a truly tailored action plan was considered beyond the scope of this project. Instead a section was dedicated to promoting the use of action plans, and encouraging individuals to visit their health professional to agree one if they didn’t have one. Rather than a dedicated family and friends section the importance of positively involving family and friends was discussed in general terms.

By the end of Phase 1 we had developed paper based versions of the web pages ready for consideration by the expert panel and for use in think aloud studies.

### Phase 2: iterative refinement of the resource contents of the website (think aloud studies and expert panel)

Eleven think aloud studies (see Table 2 for participant details) were conducted although one study (TA 08) was not completed as the website was not compatible with her type of computer which converted website text into braille (BrailleNote). Four of the 11 studies were undertaken in the participants’ own home. Three of the participants (participants 3, 4 and 10) undertook 2 studies each. Each think aloud interview covered a slightly different range of topics as the resource was developed iteratively (Table 3). Table 4 explains the nature of the changes made during this phase as a result of input from the think aloud participants and the expert panel.

**Table 3:** Think aloud studies—topics covered

	Introduction	My asthma^a^	Treatments	Asthma review	Exercise	Concerns Queries	Stress Anxiety	Action plan	4 week challenge
TA01^b^	●	●		●	●			●	
TA02		●		●		●	●	●	
TA03	●	●		●		●		●	
TA04^c^	●	● (s2)	●	●		●			●
TA05^d^	●	● (s3)	●	●	●				
TA06	●	● (s2)	●						●
TA07	●	● (s2)				●	●		●
TA08^e^									
TA09^d^	●	● (s3)						●	●
TA10^b^	●	● (s3)	●			●		●	●
TA11^c^	●	● (s2)	●	●	●	●	●		●

^a^My asthma section eventually split into 3 sections numbered s1, s2, s3. With s1 being based mainly on the contents reviewed at the first 3 think alouds before recognising need for 3 versions of this section: S1—I have never been prescribed or used a preventer inhaler; S2—I have a preventer inhaler but don’t really use it as prescribed; S3—I have a preventer inhaler and mostly use it as prescribed. ^b^TA01 and TA10 were same participant; ^c^TA04 and TA11 were same participant; ^d^TA05 and TA09 were same participant: ^e^TA08 used a Braillenote computer, which was not compatible with our software so we were unable to complete the Think Aloud study.

**Table 4:** Changes made during phase 2

Section (pages)^a^	Topics	Description of changes made
1 (13 pages)	Introduction pages^b^ Home page	Original one page introduction became 13+ page section.
		• Both TA participants and expert panel highlighted that people with asthma are well known for underestimating their asthma severity, and suggested it was important to challenge this idea right at the start and illustrate to users how this resource could benefit them.
		• First page presented user with questions designed to tease out limitations due to asthma. Then feedback provided for each question user ticked, along with tailored advice about which sections of the resource might benefit them most.
		• Subsequent pages focused on identifying lifestyle goals relevant to users.
		• Other changes included addition of a ‘landing’ page, combining links to sections to reduce the ‘buttons’ in the navigation bar from 11 down to 7, and rearranging the home page.
2 (24 pages)	My Asthma^b^	Initially just one section, but became apparent that resource needed to be more tailored, and preventer therapy use was a good method of stratifying users, so users had to choose one of three options:
		1) I have never used/been prescribed a preventer
		2) I have been prescribed a preventer but don’t really use it
		3) I mostly/always take my preventer inhaler as prescribed
		The think aloud study confirmed the contents of this section, with most changes focusing on improving readability, removing repetition and trying to achieve the right balance when explaining negative side effects versus potential benefits of inhaled steroids.
3 (14 pages)	Treatments	Organization of this section completely altered. It initially took the form of 6 pages users worked through with sideway steps for more information about different treatments.
		Section changed to have:
		• its own homepage (i.e. spoke and wheel layout) which allowed users to go directly to a treatment type without having to work through potentially irrelevant pages.
		• a visual representation of the asthma treatment ladder adapted from the BTS/Sign guidelines.
		We were unable to meet requests to have pictures of individual inhalers.
4 (21 pages)	Asthma Reviews	• Focused on modifying the language used and simplifying messages.
		• Altering layout of both individual pages and order of pages.
		• Main message was to “aim for no symptoms” and this was very well received by users.
		• Included a quiz covering what put people at risk of attacks—this was streamlined and made optional.
5 (5 pages)	Action Plans	• Altered layout and clarity of wording, and quotes added to dilute the very factual nature of the information provided.
		• Added a template to a blank action plan that users could print out and take to their health professional.
6 (17 pages)	Physical Activity	• Initially one generic section with the aim of promoting physical activity but was altered to become tailored to the individual’s activity status.
7 (18 pages)	Common concerns and queries	• Originally had 8 concerns and queries, and a further 7 were added addressing topics originally not included as were felt to be covered elsewhere, or had seemed ‘too basic’.
		• Reviewing this section served as a reminder that people quickly forget (or have never been told) even basic information about their asthma, and that having it here for those who need it was essential.
		• Another major change was the wording of questions. One user commented that questions were just statements and didn’t make it clear than scenarios were amenable to change. So for example ‘I don’t exercise because of my asthma’ was changed to ‘I don’t exercise because of my asthma. Could I?’
8 (5 pages)	Stress & Anxiety	• Received mainly positive feedback.
		• Links to online resources aimed at reducing stress and anxiety (e.g. online CBT) added.
9 (8 pages)	Take the 4 week Challenge	• This section was specifically for users who had chosen option 1 or 2 during the ‘My Asthma’ section.
		• Initially much confusion about the nature of the challenge with some users misunderstanding it completely. Thus pages were modified and more explanation added.
		• Layout of pages were altered, in particular, to make it clear that there were 4 steps to work through, and it was made clearer how you were progressing through them (e.g. colour strip across the top, which illustrated progress).
		• One of the steps to the four week challenge was to anticipate barriers to taking preventer medication regularly and consider some solutions. Template barriers and solutions were provided, and these were added to by the think aloud participants.
10	Like to stop smoking?	• This section was a link to an external site called ‘StopAdvisor’[19] and therefore not covered during the think aloud studies.
11 (1 page)	Useful info and links	• Expanded during the think aloud to include more links to online mental health resources and information about the GP exercise referral scheme.

^a^Refers to unique pages per section. Some pages are referred to in more than one section, but are only counted once here in the first section they appear

^b^All users are directed through these two sections at first login, and can optionally visit again during future sessions

Thematic analysis of the think aloud transcripts identified three main thematic categories: 1) ‘content’—the actual words on the pages, and how relevant and understandable the information was; 2) ‘layout and navigation’—the layout of pages or sections, and how easy it was to navigate around sections; and 3)‘user experience’.

**Fig. 2:**
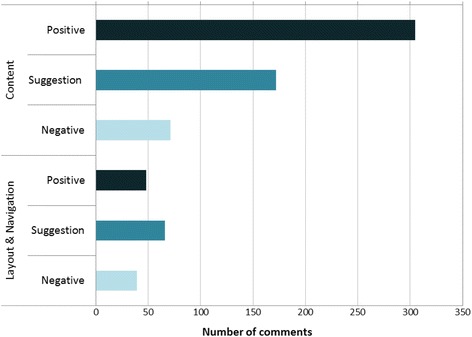
Type of comment made during think aloud studies

NVivo software allowed us to generate quantitative data from the think aloud transcripts. Fifty one percent of the comments were positive, 15 % negative and 34 % containing suggestions for improvement. This suggests that participants felt comfortable criticising or making suggestions for improvement of the website. Most comments related to the content of pages (78 %), and the majority of these were positive (56 %). In contrast, most comments about the website layout and navigation were negative (69 %) (Fig. 2). This confirmed that the ground work done in Phase 1 around content had been successful, but that greater emphasis was needed on usability and presentation issues.

### Content—making the website relevant and understandable

Participants were positive about the contents, and in particular the ‘level’ it was aimed at:

*“it’s very clear in its intention, a website to help you stay healthy and manage your asthma better that’s exactly what level I’m at, I don’t have a detailed knowledge of what I’ve got or quite what I’ve got or quite how to look after it so it’s perfect for me.” (Participant 10,TA 01)*

Users liked and identified with the key messages, for example that people with asthma should be ‘aiming for no symptoms’:

*“I like a message of you know that’s what you should be aiming for, it might not be what you get right enough but at least you should be aiming for, or aiming for it the majority of the time, you know but you can if, you know going to have relapses, but I think that that’s really good because I don’t think many people actually say that to you to be honest.” (Participant 1, TA02)*

*“That’s good to know because again I just was putting up with it like if I was, if I wasn’t being able to breathe I would just be like oh I'm just having a bad day rather than being like ‘oh I should really be on the brown inhaler to stop this from happening’,” (Participant 3, TA04)*

While there was universal agreement that quotes from patients and practice nurses were desirable within the website there was some disagreement about how they should be presented:

*“But I would give them maybe slightly more weight if they weren’t anonymous bizarrely. And it’s a real living patient that is living with asthma. And that kind of makes it more of a human.” (Participant 10, TA01)*

In the following think aloud study this point was brought up by the interviewer:

*“the quotes do you think, would you prefer to see something like female age 53 or is it not relevant? (researcher)*

*It’s not relevant to be honest because if I was twenty one and I was reading and they were fifty I would be thinking oh that doesn’t apply to me yet. The guy will be reading it and thinking oh that’s a woman thing.” (Participant 1, TA02)*

**Fig. 3:**
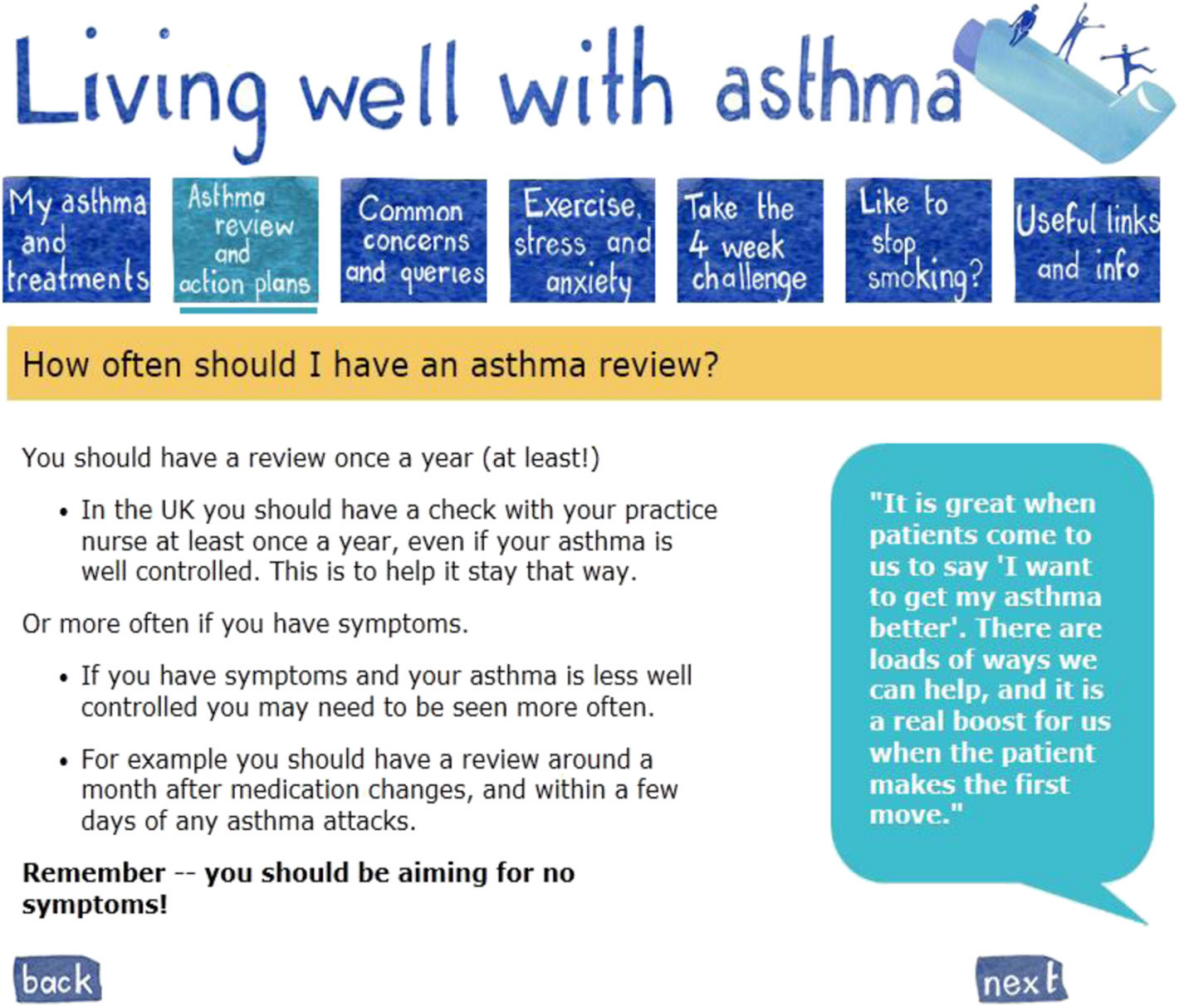
Screenshot of webpage illustrating use of quotes

Consequently, we kept quotes in the website but removed descriptions of who said them, as illustrated in Fig. 3.

While patients on the whole agreed with the information provided, the one area where there was scepticism was in regard to how approachable participants’ practices nurses were:

*“just trying to imagine sort of sitting down with my asthma nurse and saying I have a goal and this is what I want to achieve, I know what she’d say, she’d say I haven’t got time to discuss this! Let’s just stick to the tick boxes shall we?“(Participant 4, TA05)*

### Layout and navigation—making the website easy to use

The majority of the comments regarding layout were page specific such as feeling that a given paragraph was too long, and where appropriate were acted on immediately after the think aloud study in preparation for the next one. However the importance of getting the home page right was clearly important to participants and generated discussion.

*“it doesn’t quite feel like a home page, that’s maybe not helpful. I’m trying to think what the best way to, it looks the same as every other page, I don’t know if you did something different to the header or something like that.” (Participant 4, TA05)*

**Fig. 4:**
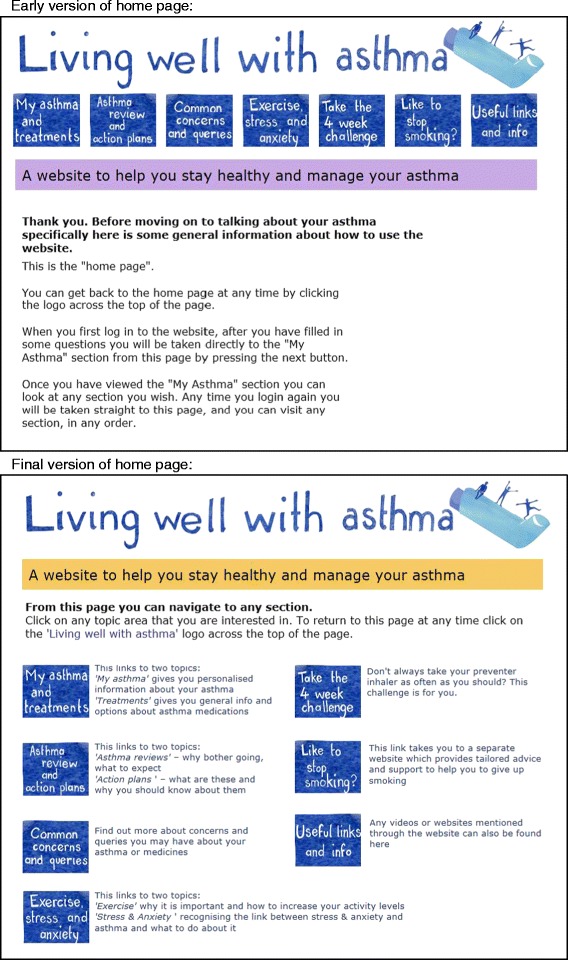
Changes made to home page during Phase 2

Therefore the home page was modified as illustrated in Fig. 4 in response to comments across the studies.

The second recurring theme related to users ‘knowing where I am’.

*“I say I might have said before maybe a little site map you are on step 3 of 9, 4 of 9 and people know where they are going.” (Participant 4, TA09)*

As a result it became more obvious which section a user was in at a given time, and within the 4 week challenge section it was made much more obvious how users progressed through the 4 stages of preparing to sign up to the ‘4 week challenge’.

### User experiences

After completing the think aloud study users were asked how they might use the website in a real life setting and what would be barriers to its sustained use. Users felt that they would have more confidence in such a resource if a health professional recommended it:

*“I guess like in my annual review, if my nurse was like oh have a look at this. Like a wee leaflet or a wee business card or something like that and just was like have a look at that.” (Participant 2, TA03)*

This finding is relevant for both future large scale RCTs, and the subsequent implementation and embedding of such a resource.

Completion of this phase resulted in the final website ready for evaluation in the RAISIN trial [23]. Table 5 describes the final contents of the resource, and further sample screenshots are provided in Additional file 2.

**Table 5:** Contents of Living Well with Asthma resource

Topic	Summary of content
Introduction pages^a^	This section encourages users to recognise whether they are putting up with symptoms unnecessarily, and introduces concepts such as goal setting and its potential benefits.
My Asthma^a^	There are three versions of this section tailored to current use of preventer therapy as chosen by the user:
	1) I have never used/been prescribed a preventer
	2) I have been prescribed a preventer but don’t really use it
	3) I mostly/always take my preventer inhaler as prescribed
	This section covers adherence and challenges negative beliefs about inhaled steroids.
Treatments	Provides information about different treatments. Links to videos to demonstrate inhaler technique and encourages users to consider whether they are on the correct ‘step’ of the asthma treatment ladder.
Asthma Reviews	Promotes attendance at asthma reviews outlining potential benefits to symptoms and quality of life. Prompts user to recognise if putting up with symptoms, and to recognise if they are at risk of asthma attacks.
Action Plans	Describes what action plans are and their potential benefits. Provides a template action plan that can also be used by practice nurses during asthma reviews in local health boards.
Physical Activity	Promotes benefits of physical activity, and challenges negative beliefs about exercising with asthma. Provides practical advice and tips to encourage users to increase their activity levels.
Common concerns and queries	Answers 15 common queries and concerns that people with asthma may have, developed from the literature, focus groups and during think aloud studies. For example:. I am worried about taking inhaled steroids long term, should I be? Why are some days better than others?
Stress & Anxiety	Promotes recognition of the role of stress on asthma, and how having asthma symptoms can lead to stress. Provides suggestions for reducing stress and anxiety.
Take the 4 week Challenge	The user is prompted to commit to taking their preventer inhaler regularly for 4 weeks. Users can choose from a list of provided ‘barriers’ to taking their inhalers and review suggested strategies or can free text their own. They may sign up to receive weekly emails during the challenge.
Like to stop smoking?	This links to an external website called ‘StopAdvisor’ [19]. This has been developed using LifeGuide software and further details are available elsewhere.
Useful info and links	This re-lists information and useful links that have been included elsewhere in the website.
Email reminders	These emails are sent every two months. They all include the RCP 3 Questions to encourage the user to assess their current control and prompt them to visit the website or see their nurse or doctor if appropriate. There are also reminders to order inhalers, or other medications (e.g. in time for hay fever season), or if going on holidays.

^a^All users are directed through these two sections at first login, and can optionally visit again during future sessions

### BCTs present in website

We incorporated 20 BCTs in our Living well with Asthma website as described in Table 6. The most commonly used BCTs were ‘information about health consequences’ and ‘demonstration of the behaviour’, followed by ‘problem solving’ and ‘instruction on how to perform a behaviour’. We also used ‘goals and planning’ as a key behavioural technique within the website.

**Table 6:** Behaviour change technique mapping of Living Well with Asthma resource

No/ Label [21]	Definition	Sections	Example within LWWA website
Goals and planning			
1.1 *Goal setting (behaviour)*	Set or agree on a goal defined in terms of the behaviour to be achieved	4 week challenge	Users commit to taking their preventer inhaler regularly for 4 weeks.
1.2 *Problem solving*	Analyse, or prompt the person to analyse, factors influencing the behaviour and generate or select strategies that include overcoming barriers and/or increasing facilitators (includes ‘Relapse Prevention*’ and ‘*Coping Planning*’*)	My asthma Concerns & queries 4 week challenge	Users are prompted to consider reasons why they find it difficult to take their inhaler regularly (choosing from a list or free texting own). Users are then presented with sample strategies to overcome identified barriers.
1.3 *Goal setting (outcome)*	Set or agree on a goal defined in terms of a positive outcome of wanted behaviour	Intro	Users are asked to identify how their asthma can negatively affect their everyday lives. They are then asked to review positive outcome goals to overcome these negative effects.
1.6 *Discrepancy between current behaviour and goal*	Draw attention to discrepancies between a person’s current behaviour (in terms of the form, frequency, duration, or intensity of that behavior) and the person’s previously set outcome goals, behavioural goals or action plans (goes beyond self-monitoring of behaviour)	Asthma Review	Asks validated questions to determine if currently putting up with asthma symptoms while believing themselves to be well controlled.
1.9 *Commitment*	Ask the person to affirm or reaffirm statements indicating commitment to change the behaviour Note: if defined in terms of the behaviour to be achieved also code 1.1, Goal setting (behaviour)	4 week challenge	Users tick three statements confirming they are committed to taking their preventer inhaler regularly for the duration of the 4 week challenge.
Social support			
3.1 *Social support (unspecified)*	Advise on, arrange or provide social support *(e.g. from friends, relatives, colleagues,’ buddies’ or staff)* or non-contingent praise or reward for performance of the behaviour*.* It includes encouragement and counselling, but only when it is directed at the behaviour	Concerns & queries	‘Where can I talk to other people about asthma’ section details and links to online forum, local support groups, and advice lines.
Shaping knowledge			
4.1 *Instruction on how to perform a behavior*	Advise or agree on how to perform the behaviour (includes ‘Skills training’)	Treatments Asthma Review Exercise	Users are given step by step instructions on how to use an inhaler correctly. This is followed up by a video demonstration.
4.3 *Re-attribution*	Elicit perceived causes of behaviour and suggest alternative explanations *(e.g. external or internal and stable or unstable)*	Concerns & queries	Describe common reasons why people with asthma put up with symptoms, illustrating that these beliefs are mistaken and providing alternative explanations for the symptoms.
Natural consequences			
5.1 *Information about health consequences*	Provide information (e.g. written, verbal, visual) about health consequences of performing the behaviour	Intro My asthma Treatments Asthma review Exercise Concerns & queries Action plans	Information provided that people who attend for regular asthma reviews have fewer symptoms and fewer asthma attacks.
5.3 *Information about social and environmental consequences*	Provide information (e.g. written, verbal, visual) about social and environmental consequences of performing the behaviour	Asthma review Exercise	Information provided that people who attend for regular asthma reviews have fewer days off school and work, and fewer limitations in activities.
5.6 *Information about emotional consequences*	Provide information (e.g. written, verbal, visual) about emotional consequences of performing the behaviour	Concerns & queries	People with asthma describe feeling embarrassed or ashamed taking inhalers in public. Information provided to overcome these concerns and increase confidence about using medications in public.
Comparison of behaviour			
6.1 *Demonstration of the behaviour*	Provide an observable sample of the performance of the behaviour, directly in person or indirectly e.g. via film, pictures, for the person to aspire to or imitate (includes ‘Modelling’).	My asthma Treatments Asthma review Exercise Action plans	Quotes for adults with asthma demonstrating how their lives changed for the better when they started taking their inhalers regularly.
6.2 *Social comparison*	Draw attention to others’ performance to allow comparison with the person’s own performance	My asthma Concerns & queries	In those who have identified that their asthma affects their work they are advised that this is the case with up to 40 % of people with asthma.
6.3 *Information about others’ approval*	Provide information about what other people think about the behaviour. The information clarifies whether others will like, approve or disapprove of what the person is doing or will do	Asthma review	Quote from practice nurse praising people who proactively attend for asthma reviews.
Associations			
7.1 *Prompts/cues*	Introduce or define environmental or social stimulus with the purpose of prompting or cueing the behaviour. The prompt or cue would normally occur at the time or place of performance	4 week challenge Emails	Users who sign up to the 4 week challenge are sent weekly emails to remind them of the challenge and prompt them to continue.
Repetition and substitution			
8.2 *Behavior substitution*	Prompt substitution of the unwanted behaviour with a wanted or neutral behaviour	Exercise	Users are provided with sample strategies to increase their levels of physical activity such as walking to the shops rather than taking the car, or giving up a TV programme for a dance class.
8.3 *Habit formation*	Prompt rehearsal and repetition of the behaviour in the same context repeatedly so that the context elicits the behaviour	4 week challenge	Strategies for prompting users to remember to take inhalers are suggested such as using them at the same time as teeth brushing or the evening meal.
Comparison of outcomes			
9.1 *Credible source*	Present verbal or visual communication from a credible source in favour of or against the behaviour	Exercise	Bradley Wiggins quote describing how asthma doesn’t stop him exercising.
Antecedents			
12.5 *Adding objects to the environment*	Add objects to the environment in order to facilitate performance of the behaviour.	4 week challenge	Strategies for prompting users to remember to take inhalers are suggested such having an extra inhaler at work, if they regularly forget their morning dose.
Self-belief			
15.1 *Verbal persuasion about capability*	Tell the person that they can successfully perform the wanted behaviour, arguing against self-doubts and asserting that they can and will succeed	Exercise (external video)	Users are directed to a video which promotes the message that anyone regardless of health status and fitness levels can successfully increase their levels of physical activity.

## Discussion

We have demonstrated the feasibility of developing an evidence-based, theory guided, user friendly behaviour change intervention in the form of Living well with Asthma—a website to support self-management in adults with asthma. We have been guided by the MRC Framework on developing and evaluating complex interventions, and as a result directed much effort to the key, yet often overlooked, planning stages [5, 18]. We undertook recommended key tasks to guide our development methods [5], see Table 1, through: synthesis of empirical evidence, using expert knowledge and experience, and incorporating theoretical concepts with end user input, to produce an evidence based behaviour change website. Our evidence synthesis highlighted that self-management of asthma is an underused, yet proven, treatment strategy [7] and that people with asthma frequently do not use therapies optimally, tolerate high levels of symptom burden, and do not attend asthma reviews or make use of asthma action plans. This underestimation of symptoms and overestimation of control is a barrier to making use of available therapies. Those who do recognise they are experiencing asthma symptoms often do not adhere to therapies, often due to perceived misunderstandings around medication side-effects, or lack of perceived benefits to using asthma action plans. This analysis provided us with pathways of how a behaviour change intervention might work, focussing on behaviour change concepts recommended in the literature we developed a list of features which a website should have. This list was iteratively modified with input from end users and an expert panel, until a draft of proposed web pages had been developed. These were then gradually converted to working interactive webpages and refined over 10 think aloud studies, to lead to the final website which is being evaluated in the RAISIN trial [23]. Our BCT mapping exercise demonstrates that the resource makes use of multiple BCTs, a strategy which in some health domains has been associated with increased effect sizes [37]. In particular we use goals and planning as a key behavioural technique, which has been shown to be efficacious in asthma [1].

### Strengths

This study followed recommended processes for developing complex evaluations, and was undertaken by a multidisciplinary team with a range of essential skills, knowledge and experience (including behaviour change theory and implementation theory). A key strength of this resource is in its co-design with potential end users, who had opportunity for input both at the early development planning stages in the form of focus groups, and also towards the end where their input via think aloud studies was invaluable in improving the usability of the resource, in line with the Person Based Approach [15]. The use of LifeGuide software allowed for a streamlined and iterative process of website development where the researcher taking the think aloud studies could modify the website directly following think aloud studies, or from feedback from the expert panel. Most computer programmers do not have a background in healthcare, and therefore removing the need to communicate user feedback to a programmer by using LifeGuide made the process far more efficient.

### Limitations

In the focus groups we invited both practice nurses and adults with asthma which could be construed as a limitation. However there are advantages to bringing together a diverse group of participants and we felt this was the case here [40]. This can maximise the exploration of different perspectives, which was pertinent here where differences in health professional and patient opinion is a recognised barrier to optimal uptake of self-management practices [31].

The adults with asthma participating in the focus groups and think aloud studies had more severe asthma and were on more treatments than typical primary care patients. This is almost certainly because of them being recruited through their participation in asthma advocacy organisations (Asthma UK). We managed this by tempering the suggestions and feedback from these end users with the practical experience of the respiratory physicians and GPs on the expert panel and the practice nurses present in the focus groups. In future studies concentrating recruitment to end users more typical of a primary care population would be worthwhile, although may be difficult.

The same researcher who developed the website, also undertook the think aloud studies. While this had benefits in terms of speed of modifying the resource, we were concerned that participants in the think aloud studies may not have felt comfortable criticising the resource openly in the presence of the person who was also developing it. In order to counter this it was explained that it was easy to make changes with the LifeGuide software and those critical comments were often the most helpful. Exploring the scope of this limitation by counting negative comments was useful, as the high proportion of negative comments or suggestions for improvements suggests that participants did feel comfortable being critical of the website.

### Future considerations

The ultimate aim of following the updated MRC guidance on the development and evaluation of complex interventions is to reduce the number of interventions which are developed, but don’t translate into everyday use, and avoiding costly large RCTs which due to unforeseen circumstances are unable to answer the research question posed [18]. The iterative methods of development used here should minimise this risk, and the ongoing RAISIN pilot RCT should allow for meaningful estimates of effect sizes and recruitment and retention rates for any future full scale randomised controlled trial. A qualitative evaluation, involving participant interviews and using NPT to guide analysis, is embedded in the RAISIN trial and will provide rich data on the how the intervention can be improved and its future implementability in the real world [24].

## Conclusion

We have developed a resource which our preliminary usability testing suggests is relevant and usable by its target audience. We have outlined the key steps undertaken which included synthesis of knowledge and experience from our expert panel, with a broad exploration of the literature, overarching use of appropriate theory (behaviour change and implementation) and also with input from potential stakeholders (adults with asthma and practice nurses) from an early planning stage. Such methods are rarely fully detailed in the literature and thus the description of this process should be of interest to the growing cadre of researchers developing digital interventions. This paper demonstrates how data from a wide range of sources can directly and practically influence the contents of such a self-management website.

## Additional files

Additional file 1:
**Rationale behind choosing the contents for Living Well with Asthma resource.**
Additional file 2:
**Additional sample screenshots from Living Well with Asthma.**

## Abbreviations

AAP: Asthma Action Plans
ACQ: Asthma Control Questionnaire
BCT: Behaviour Change Technique
HP: Health Professional
ICT: Information and Communication Technology
IQR: Interquartile Range
ISRCTN: International Standard Randomised Controlled Trial Number
MRC: Medical Research Council
NPT: Normalization Process Theory
PEF: Peak Expiratory Flow
RAISIN: Randomized Trial of an Asthma Internet Self-Management Intervention
RCP 3Q: Royal College of Physicians 3 Questions
RCT: Randomized Controlled Trial
SIMD: Scottish Index of Multiple Deprivation
TA: Think aloud
WP: Work package
