# Supplementary material for: Details of development of the resource for adults with asthma in the RAISIN (randomized trial of an asthma internet self-management intervention) study
Source: BMC Med Inform Decis Mak. 2015 Jul 28;15:57. doi: 10.1186/s12911-015-0177-z (PMC4517557; doi:10.1186/s12911-015-0177-z)
Supplement: Additional file 1: — Rationale behind choosing the contents for Living Well with Asthma resource. [file 12911_2015_177_MOESM1_ESM.pdf]

Additional File 1. Intervention Planning

Aim of intervention: reduce burden of symptoms and increase QOL through supporting use of medications.

Why is this important? See table 1.

Methods: Statements relating to adherence specifically were extracted from a previously undertaken literature review. A relevant feature of the proposed website was attributed to the statement, resulting in a 'suggested component' to be a feature of the website. This led to the production of tables showing barriers and enablers from the literature. Two articles were of specific relevance and these were looked at in detail individually, and tables relevant to their findings also produced in the same way as described above (GINA guidelines[1], chapter 4, and a review of adherence[2]. Finally analysis of the two focus groups provided information for a further set of tables describing identified barriers and enablers. Finally the information relevant to each component was grouped together to produce a table for each, illustrating the source of the evidence.

Component: Info page – on medication & side-effects with suggestions of alternatives to avoid these side-effects		
**Source**	**Finding relevant to adherence**	**Suggested feature of website.**
Asthma literature review	People with asthmas beliefs about medications can impact on adherence.[1, 3](E.g. effectiveness, tolerance, fears of side-effects)	Provide information to challenge beliefs both facts and experiences
GINA guidelines chapter 4, component 1 – key components of successful asthma education programme.[1]	• Potential side-effects	Information describing side effects and possible alternative treatments to minimise them
	• Use of inhaler device<br>• Difference between relievers and preventers	Info to illustrate different inhalers, and inhaler technique, combined with videos demonstrating technique
Adherence review[2]:<br>DiMatteo MR et al.<br>*Health Psychology Review* 2012; 6(1):74-91.	An individual's beliefs about the value of the treatment (i.e. likely risks, benefits and efficacy) and their confidence that practical barriers to adherence can be overcome are also meaningful in influencing motivation to adhere.	Information section about medications – risks, benefits.<br>Quotes illustrating positive experience of patients who started taking medication and then felt better for example
Focus group	Problems with medications (side-effects/inhaler technique) as a barrier to adherence could be overcome by acknowledging potential issues with advice about alternatives.	Information section about medications<br>Provide examples of identified barriers to adherence from literature to help user identify any relevant to self with potential strategies to overcome.

Component: Info page – benefits of an AAP		
**Source**	**Finding relevant to adherence**	**Suggested feature of website.**
Asthma literature review	People with asthma with a new diagnosis lacked confidence in using action plans.[4]	Illustrate benefits & low risk of harms
	People with asthma feel that action plans are not relevant to their situation.[5]<br>*Those described as compliant (taking optimal doses of both reliever and preventer) felt action plans do not acknowledge their own experience, irrelevant<br>* Those described as non compliant felt that action plans could be useful for people with "more serious" or "proper" asthma.	Provide info to illustrate benefits of asthma action plan

Component: Info page – how to use an AAP, including quotes/videos where applicable, incorporating the role of goals.		
**Source**	**Finding relevant to adherence**	**Suggested feature of website.**
Asthma literature review	People with asthma with a new diagnosis lacked confidence in using action plans.[4]	Provide information about how to use action plans<br>Provide examples of using AAP use, quotes aiming to increase confidence
	Guided self management with asthma plan (including health professional review improves asthma related outcomes.[6]	Information pages about how to self manage e.g. self monitor, use an AAP, role of goals in self management
	Self management education improves action plan use.[7]	Info page about how to use an action plan<br>Provide examples of using AAP, quotes aiming to increase confidence in their use
Focus group	Provide information about how to self manage to facilitate adherence.	Info pages – how to use AAPs and benefits of using them.

Component: Info page - challenge attitude (denial) and illustrate benefits of accepting diagnosis, and taking medications. Using quotes where applicable.		
**Source**	**Finding relevant to adherence**	**Suggested feature of website.**
Asthma literature review	More holistic approach to actions plans – 'living with asthma plan'.[8, 9]	Relates to tone of the intervention – make relevant to user. Discuss benefits in terms of beneficial impact on life, family, rather than medical issues such as hospitalisations.
GINA guidelines chapter 4, component 1 – key components of successful asthma education programme.[1]	• Discussion of expectations	Explain can aim for minimum symptoms, and minimal impact on day to day life, provide sample 'goals' for patients to select. Provide AAPs to facilitate goal achievement
	• Prevention of symptoms and attacks	Info illustrating risks of poor adherence on symptoms and exacerbation risk.
	• Acceptance this is continuing processes	Encourage acceptance of diagnosis by illustrating benefits of taking meds to QOL, activities, general life.
	• Expression of fears and concerns	Provide examples of fears and concerns from literature and focus groups which user may identify with. Use of quotes and info to challenge negative beliefs or attitudes, particularly around fears and concerns about medications.
Adherence review[2]:<br>DiMatteo MR et al.<br>*Health Psychology Review* 2012; 6(1):74-91.	An individual's beliefs about the value of the treatment (i.e. likely risks, benefits and efficacy) and their confidence that practical barriers to adherence can be overcome are also meaningful in influencing motivation to adhere.	Provide examples of identified barriers from literature to help user identify any relevant to self. List provided in figure 4.1-4 GINA guidelines[1] page and potential strategies to overcome. E.g. if recognise a complicated regime contributing then encourage discussion with health professional.<br>Quotes illustrating positive experience of patients who started taking medication and then felt better for example, and examples of how barriers to adherence can be overcome.
	Individuals who believe that consequences of non-adherence are severe are more likely to be adherent than those who believe that consequences less serious, and that loss framing messages are slightly more effective than gain-framed messages. (However, evidence that when preventative behaviours are the target , as here, gain framing more effective.)[10]	Information will be presented to promote the continuation of a healthy symptom free life, rather than the threat of e.g. hospitalisation to encourage adherence. This relates to general tone of intervention message.
Focus group	Taking inhaler is admission to individual that they have asthma.	Encourage acceptance of diagnosis by illustrating benefits of taking meds to their QOL, activities and general life.

Component: Info – common barriers to adherence and suggestions to overcome		
**Source**	**Finding relevant to adherence**	**Suggested feature of website, specific to component.**
Asthma literature review	Having the same type of inhaler for reliever and preventer improves outcomes of asthma control and exacerbation rate.[11]	Help patients to identify barriers to taking meds and if complicated regime contributing then encourage discussion with health professional
Adherence review[2]: DiMatteo MR et al. *Health Psychology Review* 2012; 6(1):74-91.	People with asthma must have the tools and strategies necessary and must have the capacity to overcome barriers to adherence – so important task for health professional is help patient identify and overcome barriers to adherence.	Provide examples of identified barriers from literature to help user identify any relevant to self. E.g. if recognise a complicated regime contributing then encourage discussion with health professional.
	A complex treatment regime is one of the most consistent barriers to successful adherence.	Help patients to identify barriers to taking med. If complicated regime contributing then encourage discussion with health professional.
	People with asthma must have the tools and strategies necessary and must have the capacity to overcome barriers to adherence – so important task for health professional is help patient identify and overcome barriers to adherence.	Provide examples of identified barriers from literature to help user identify any relevant to self. E.g. if recognise a complicated regime contributing then encourage discussion with health professional.
	An individual's beliefs about the value of the treatment (i.e. likely risks, benefits and efficacy) and their confidence that practical barriers to adherence can be overcome are also meaningful in influencing motivation to adhere.	Provide examples of identified barriers from literature to help user identify any relevant to self. List provided in figure 4.1-4 GINA guidelines[1] page and potential strategies to overcome. E.g. if recognise a complicated regime contributing then encourage discussion with health professional.
Focus group	Problems with medications (side-effects/inhaler technique) as a barrier to adherence could be overcome by acknowledging potential issues with advice about alternatives.	Provide examples of identified barriers to adherence from literature to help user identify any relevant to self, and potential strategies to overcome.

Component: Info page – how to self monitor & illustrate the benefits of self monitoring.		
**Source**	**Finding relevant to adherence**	**Suggested feature of website.**
Asthma literature review	People with asthma are poor at recognising deterioration in asthma symptoms, and therefore unable to act appropriately.[4]	Provide info how to self monitor<br>Provide info to show benefits of self monitoring
	Guided self management with asthma plan (including health professional review improves asthma related outcomes.[6]	Information pages about how to self manage e.g. self monitor, use an AAP, role of goals in self management<br>Provide action plans
	Self monitoring in order to recognise loss of asthma control is a grade A recommendation within the BTS/SIGN guidelines.[12].	Info about how to monitor self for deterioration in symptoms
	Weekly monitoring may be sufficient in those with well or partly controlled asthma, and that this could safely become less frequent once good control is achieved.[13]	Info about how to monitor self for deterioration i.e. symptoms or via PEF monitoring, and how often to monitor.
GINA guidelines chapter 4, component 1 – key components of successful asthma education programme.[1]	• Signs that suggest asthma is worsening and actions to take.<br>• Monitoring control of asthma.	Info about how to monitor self for deterioration in symptoms.
Adherence review[2]: DiMatteo MR et al. *Health Psychology Review* 2012; 6(1):74-91.	Multifaceted approaches work best involving combinations of strategies such as providing information and reminders, simplifying behaviour required, practicing ongoing assessment, counselling, self monitoring and providing reinforcements.	Intervention will utilise combination of strategies: information provision, tools for self monitoring, reminder emails
Focus group	Provide information about how to self manage to facilitate adherence.	Info pages – how to self monitor recognising deteriorating symptoms

Component: Info page – benefits of, and getting the most out of, the annual review

Source	Finding relevant to adherence	Suggested feature of website.
Asthma literature review	Goal setting has been a component of successful interventions in asthma, with patient centred goals described.[14]	Encourage user to consider goals to discuss at annual review.
	Guided self management with asthma plan (including health professional review improves asthma related outcomes.[6]	Information pages about role of annual review.
	Shared decision making (incorporating patient goals and preferences into the consultation) improved adherence to medication and clinical outcomes.[15]	Encourage user to consider goals to discuss at annual review.
GINA guidelines chapter 4, component 1 – key components of successful asthma education programme.[1]	• Development of a partnership between patient and health professional.<br>• Sharing of information.	Provide info about benefits of attending for health professional review.
Adherence review[2]:<br>DiMatteo MR et al.<br>*Health Psychology Review* 2012; 6(1):74-91.	A complex treatment regime is one of the most consistent barriers to successful adherence.	Help patients to identify barriers to taking meds. If complicated regime contributing then encourage discussion with health professional.
	Mental health issues represent another common barrier to successful adherence, and health professionals should assess for the presence of such issues	Point out this can be a barrier and provide links to relevant websites e.g www.glasgowsteps.com with advice to discuss with health professional.
	When individuals are adequately informed, they are better able to share in the decisions that affect their health, and are more committed to regimes that they have had a part in choosing.	Set up reminder email prior to date of due annual review, suggesting user visits website prior to annual review. Info about what to expect from the annual review and how to get the most from it.
Focus group	Participants keen to have face to face contact as part of the asthma review.	Information about getting the most of the annual review.

Component: Info page – impact of non asthma issues

Source	Finding relevant to adherence	Suggested feature of website.
Adherence review[2]:<br>DiMatteo MR et al.<br>*Health Psychology Review* 2012; 6(1):74-91.	Mental health issues represent another common barrier to successful adherence, and health professionals should assess for the presence of such issues	Point out this can be a barrier and provide links to relevant websites e.g www.glasgowsteps.com or advice to discuss with health professional.

Component: Info provided must be consistent with that provided by health care professionals and relevant charities.

Source	Finding relevant to adherence	Suggested feature of website, specific to component.
Adherence review[2]:<br>DiMatteo MR et al.<br>*Health Psychology Review* 2012; 6(1):74-91.	There is evidence that during the medical visit physicians consistently omit critical elements of information regarding medication use, thus contributing to non-adherence.	Content of website to be consistent with guideline recommendations, so will mirror what is discussed with health professionals.
Focus group	Length of time between annual reviews – difficult to retain the information.	Provide info which mirrors that discussed during annual review, and can be revisited by user at any time.
	Website to bridge the gap between asthma reviews by being source of information, and reminders (e.g. hay fever season, flu jab due).	Information will be available at all times via website, and will mirror that discussed in annual asthma reviews
	Website being recommended by nurses during asthma reviews.	Information will mirror that discussed in annual asthma reviews.<br>Website informed by inclusion of practice nurses during focus groups studies

Component: Tool for monitoring e.g. ACT or ACQ or PEF diary/calculator		
**Source**	**Finding relevant to adherence**	**Suggested feature of website, specific to component.**
Asthma literature review	People with asthma overestimate their control and tolerate unnecessary symptoms[16]:	Provide tool to assess control/symptoms e.g. ACT, ACQ
	People with asthma are poor at recognising deterioration in asthma symptoms, and therefore unable to act appropriately.[4]	Provide tools for self monitoring e.g. ACT, ACQ with resultant action plans to ensure appropriate action taken.
	People with asthma alter medications inappropriately in response to a perceived deterioration in symptoms.[17]	Tool to aid assessment of current control either by PEF or symptom score
	Self monitoring in order to recognise loss of asthma control is a grade A recommendation within the BTS/SIGN guidelines.[12]	Info about how to monitor self for deterioration in symptoms<br>Tool to use to assess current control either symptom score or PEF
	Weekly monitoring may be sufficient in those with well or partly controlled asthma, and that this could safely become less frequent once good control is achieved.[13]	Info about how to monitor self for deterioration i.e. symptoms or via PEF monitoring, and how often to monitor.
GINA guidelines chapter 4, component 1 – key components of successful asthma education programme.[1]	• Signs that suggest asthma is worsening, and actions to take<br>• Monitoring control of asthma<br>• How and when to seek medical attention	Provision of tools to facilitate self monitoring.
	• Prevention of symptoms and attacks	Tool to establish current level of control, and advice on action to take.
Adherence review[2]:<br>DiMatteo MR et al.<br>*Health Psychology Review* 2012; 6(1):74-91.	Multifaceted approaches work best involving combinations of strategies such as providing information and reminders, simplifying behaviour required, practicing ongoing assessment, counselling, self monitoring and providing reinforcements.	Intervention will utilise combination of strategies: information provision, tools for self monitoring, reminder emails
Focus group	Not being prepared for flare – either unexpected worsening of symptoms, or for time of year when symptoms regularly more problematic.	Provide tool to establish if control is poor e.g. ACT, ACQ<br>Use of reminder emails e.g. in spring in case worsens with pollen.
	Provide information about how to self manage to facilitate adherence.	Info pages – how to self monitor recognising deteriorating symptoms, how to use AAPs and benefits of using them. How to recognise barriers to adherence.<br>Tools – to monitor self either with symptoms (ACQ or ACT) or via PEF(via calculator or diary)<br>Tools to promote adherence e.g. using goal setting and action plans.

Component: Email reminders		
**Source**	**Finding relevant to adherence**	**Suggested feature of website, specific to component.**
Asthma literature review	Guided self management with asthma plan (including health professional review improves asthma related outcomes.[6]	Email reminders about attending for health professional review
	Shared decision making (incorporating patient goals and preferences into the consultation) improved adherence to medication and clinical outcomes.[15]	Email reminders to encourage viewing of website prior to annual review
GINA guidelines chapter 4, component 1 – key components of successful asthma education programme.[1]	• Development of a partnership between patient and health professional.<br>• Sharing of information.	Email reminders to facilitate attendance at review
	• Person then requires regular supervision, revision, reward, reinforcement	Occasional email reminders to think about recent control e.g RCP 3 questions, if haven't logged on for a set time period. 'Congratulations' message if consistently demonstrates good control when using assessment tool.<br>If achieve preset goal, then receive email or message recognising this.
Adherence review[2]:<br>DiMatteo MR et al.<br>*Health Psychology Review* 2012; 6(1):74-91.	Multifaceted approaches work best involving combinations of strategies such as providing information and reminders, simplifying behaviour required, practicing ongoing assessment, counselling, self monitoring and providing reinforcements.	Intervention will utilise combination of strategies: information provision, tools for self monitoring, reminder emails
	It is crucial to assess, and to regularly track the continuing adherence status of individual patients as it is one of the best ways to estimate future behaviour.	Occasional email reminders to think about recent control e.g RCP 3 questions, if haven't logged on for a set time period.
	When individuals are adequately informed, they are better able to share in the decisions that affect their health, and are more committed to regimes that they have had a part in choosing.	Set up reminder email prior to date of due annual review, suggesting user visits website prior to annual review.
Focus group	Not being prepared for flare – either unexpected worsening of symptoms, or for time of year when symptoms regularly more problematic.	Use of reminder emails e.g. in spring in case worsens with pollen.
	Website to bridge the gap between asthma reviews by being source of information, and reminders (e.g. hay fever season, flu jab due).	Email reminders
	Providing means to track use of medications, or flag up need to order meds in.	Email reminders

Component: Provide links to relevant websites		
**Source**	**Finding relevant to adherence**	**Suggested feature of website, specific to component.**
Adherence review[2]:<br>DiMatteo MR et al.<br>*Health Psychology Review* 2012; 6(1):74-91.	Mental health issues represent another common barrier to successful adherence, and health professionals should assess for the presence of such issues	Point out this can be a barrier and provide links to relevant websites e.g www.glasgowsteps.com or advice to discuss with health professional.

**Component: Make visually appealing and accessible as possible e.g. graded Info, videos, images, option to print pages relevant to the individual**		
**Source**	**Finding relevant to adherence**	**Suggested feature of website, specific to component.**
Asthma literature review	Impaired literacy is associated with reduced asthma knowledge and improper inhaler use,[18] reduced aural literacy is associated with poorer asthma control measured by nights with symptoms.[19]	Provide information in a graded way, where user can determine depth of information required. Use images, videos.
GINA guidelines chapter 4, component 1 – key components of successful asthma education programme.[1]	• Use of inhaler devices<br>• Difference between relievers and preventers	Info to illustrate different inhalers, and inhaler technique, combined with videos demonstrating technique
Adherence review[2]:<br>DiMatteo MR et al.<br>*Health Psychology Review* 2012; 6(1):74-91..	Individuals are only capable of doing what they clearly understand; unintentional non-adherence is often rooted in failures at this stage of the process.	User can determine depth of information by presenting info in graded form<br>Provide information which has undergone user review (think aloud studies) to optimise users ability to understand it.
	There is evidence that during the medical visit physicians consistently omit critical elements of information regarding medication use, thus contributing to non-adherence.	Providing alternative comprehensive source of information available 24/7 via a website.
	Several meta-analysis have highlighted the importance of tailoring to obtain optimum effectiveness	User can determine depth of information by presenting info in graded form.
	Providing information to individuals is essential, but not sufficient to ensure adherence. More information leads to improved recall (but patients can become overwhelmed) and better outcomes when physicians assess patients' recall.	Provide a 'print this page' button so that users can print off particular pages that are relevant to them.
	When individuals understand clearly and remember what they are asked to do, they are much more likely to do it.	User can determine depth of information by presenting info in graded form.<br>Provide information which has undergone user review (think aloud studies) to optimise users ability to understand it.
Focus group	Length of time between annual reviews – difficult to retain the information.	Provide info which mirrors that discussed during annual review, and can be revisited by user at any time.<br>'Print this page option'
	Staggering the available information to be relevant as possible	User can determine depth of information by presenting info in graded form.<br>Provide information which has undergone user review (think aloud studies) to optimise users ability to understand it.
	Website to bridge the gap between asthma reviews by being source of information, and reminders (e.g. hay fever season, flu jab due).	Information will be available at all times via website, and will mirror that discussed in annual asthma reviews
	Making the information fun and attractive.	Provide information which has undergone user review (think aloud studies) to optimise users ability to understand it.<br>Use images and videos where relevant.

Component: Asthma action plan (AAP)		
**Source**	**Finding relevant to adherence**	**Suggested feature of website, specific to component.**
Asthma literature review	Health professionals don't always offer action plans[4, 5, 20]	Provide alternative means of accessing action plan via freely available website
	People with asthma are poor at recognising deterioration in asthma symptoms, and therefore unable to act appropriately.[4]	Provide tools for self monitoring e.g. ACT, ACQ with resultant action plans to ensure appropriate action taken.
	People with asthma alter medications inappropriately in response to a perceived deterioration in symptoms.[17]	Provide action plan to guide medication alteration
	Health professionals belief that actions plans only suitable for certain patients – e.g well educated with well controlled asthma. [5]	Provide alternative means of accessing action plan via freely available website
	Guided self management with asthma plan (including health professional review improves asthma related outcomes.[6]	Provide action plans
GINA guidelines chapter 4, component 1 – key components of successful asthma education programme.[1]	• Monitoring control of asthma<br>• How and when to seek medical attention	Provision of AAP to guide medication changes, changes to monitoring frequency, or to suggest health professional review.
	• Prevention of symptoms and attacks	Tool to establish current level of control, and advice on action to take.
	• Person then requires a written asthma action plan	Provide asthma action plans, this can relate to medication changes, frequency of self-monitoring, when to seek input from health professional.
Focus group	Provide information about how to self manage to facilitate adherence.	Tools to promote adherence e.g. using goal setting and action plans.

Component: Menu of template goals with associated action plans to achieve goal		
**Source**	**Finding relevant to adherence**	**Suggested feature of website, specific to component.**
Asthma literature review	Goal setting has been a component of successful interventions in asthma, and patient centred goals decribed.[14]	Provide menu template goals (lifestyle rather than medication)<br>Provide action plans to facilitate goal achievement (not just medication related, could be exercise, self monitoring, stopping smoking)
	More holistic approach to actions plans – 'living with asthma plan'.[8, 9]	Relates to tone of the intervention – template goals provided with relevant advice to achieve goal relevant to individual.
	Shared decision making (incorporating patient goals and preferences into the consultation) improved adherence to medication and clinical outcomes.[15]	Provide template goals (lifestyle rather than medication) with assoc action plans to help achieve goals.
	Qualitative work shows that from a patients perspective that while these goals[no night time cough etc] are acknowledged they are mediatory and patients prefer to consider end state lifestyle goals such 'playing football again' or having 'a normal life'. [21]	Explain can aim for minimum symptoms, and minimum impact of day to day life, provide sample 'goals' for patients to select which are relevant to own life, based on this qualitative work.
GINA guidelines chapter 4, component 1 – key components of successful asthma education programme.[1]	• Discussion of expectations	Explain can aim for minimum symptoms, and minimal impact on day to day life, provide sample 'goals' for patients to select. Provide AAPs to facilitate goal achievement
Focus group	Provide information about how to self manage to facilitate adherence.	Tools to promote adherence e.g. using goal setting and action plans.

Component: Info -encourage positive involvement of family/friends in management		
**Source**	**Finding relevant to adherence**	**Suggested feature of website, specific to component.**
Asthma literature review	Role of social relationships can negatively impact on people with asthmas ability to self manage, e.g. perceived 'nagging' from family members to take medication, over reactions, or indifference.[22]	Provide page aimed at family/friends about how can support the person with asthma to manage their asthma as well as possible
	Positive social relationships e.g. helpful reminders to take medications.[22]	Provide info to illustrate potential beneficial role of family friends, with page targeted to family or friends.
Adherence review[2]: DiMatteo MR et al. *Health Psychology Review* 2012; 6(1):74-91.	Cultural norms, family members and friends also strongly influence patients' decisions about health actions – particularly through their goals and intentions – and adherence to treatment is no exception.	Provide info page targeted at family/friends to encourage positive influence on adherence.

Component: Tailored asthma action plan		
**Source**	**Finding relevant to adherence**	**Suggested feature of website, specific to component.**
Asthma literature review	People with asthma find action plans are not relevant or useful to their own situation[9]	Tailor asthma action plans (e.g. to severity, experience, goals) in the context of living with asthma plan
	People with asthma feel that action plans are not relevant to their situation.[5]<br>*Those described as compliant (taking optimal doses of both reliever and preventer) felt action plans do not acknowledge their own experience, irrelevant<br>* Those described as non compliant felt that action plans could be useful for people with "more serious" or "proper" asthma.	Tailor action plan based on severity
	Several meta-analysis have highlighted the importance of tailoring to obtain optimum effectiveness	Action plans can be tailored where possible.

Component: Diary tool for keeping track of medication used		
**Source**	**Finding relevant to adherence**	**Suggested feature of website, specific to component.**
Focus group	Difficult to keep track of what medication has been used, and need for ordering more. Would like means to track medication use, particularly reliever medication.	Provide a diary tool for keeping track of medication use.

Component: Facilitating recall - self test quizzes		
**Source**	**Finding relevant to adherence**	**Suggested feature of website, specific to component.**
Adherence review[2]: DiMatteo MR et al. *Health Psychology Review* 2012; 6(1):74-91.	Providing information to individuals is essential, but not sufficient to ensure adherence. More information leads to improved recall (but patients can become overwhelmed) and better outcomes when physicians assess patients' recall.	Provide optional self test quizzes
	When individuals understand clearly and remember what they are asked to do, they are much more likely to do it.	Self test quizzes may aid recall, as will option to print specific pages

AAP – Asthma action plan (this can refer to a plan advising about altering medications, monitoring regimes, when to seek health professional review); ACQ – Asthma control questionnaire; ACT – Asthma Control Test; PEF – peak expiratory flow; RCP 3Q – Royal College Physicians 3 Questions (to assess control)

References

1. **Global Strategy for Asthma Management and Prevention 2011 (Update)** [http://www.ginasthma.org/uploads/users/files/GINA_Report2011_May4.pdf]
2. DiMatteo MR, Haskard-Zolnierek KB, Martin LR: **Improving patient adherence: a three-factor model to guide practice**. *Health Psychology Review* 2012, **6**(1):74-91.
3. Partridge MR, Dal Negro RW, Olivieri D: **Understanding patients with asthma and COPD: insights from a European study**. *PrimCare RespirJ* 2011, **20**(3):315-323, 317.
4. Douglass J, Aroni R, Goeman D, Stewart K, Sawyer S, Thien F, Abramson M: **A qualitative study of action plans for asthma**. *BMJ* 2002, **324**(7344):1003.
5. Jones A, Pill R, Adams S: **Qualitative study of views of health professionals and patients on guided self management plans for asthma**. *BMJ* 2000, **321**(7275):1507-1510.
6. Gibson PG, Powell H, Wilson A, Abramson MJ, Haywood P, Bauman A, Hensley MJ, Walters EH, Roberts JJ: **Self-management education and regular practitioner review for adults with asthma [Systematic Review]**. *Cochrane Database of Systematic Reviews* 2002, **3**.
7. Ring N, Malcolm C, Wyke S, Macgillivray S, Dixon D, Hoskins G, Pinnock H, Sheikh A: **Promoting the use of Personal Asthma Action Plans: a systematic review.** *Primary Care Respiratory Journal* 2007, **16**(5):271-283.
8. Ring N, Pinnock H, Wilson C, Hoskins G, Jepson R, Wyke S, Sheikh A: **Asthma plans: Understanding what we mean. Linguistic analysis of terminology as used in published texts.** *Primary Care Respiratory Journal* 2011, **20**(2):170-177.
9. Ring N, Jepson R, Hoskins G, Wilson C, Pinnock H, Sheikh A, Wyke S: **Understanding what helps or hinders asthma action plan use: A systematic review and synthesis of the qualitative literature**. *Patient Education and Counseling* 2011, **85**(2):e131-e143.
10. Polak L: **Communicating risk to patients and the public**. *BrJ GenPract* 2012, **62**(598):240.
11. Price D, Chrystyn H, Kaplan A, Haughney J, Roman-Rodriguez M, Burden A, Chisholm A, Hillyer EV, von ZJ, Ali M *et al*: **Effectiveness of same versus mixed asthma inhaler devices: a retrospective observational study in primary care**. *Allergy Asthma ImmunolRes* 2012, **4**(4):184-191.
12. British Thoracic Society: **British Guideline on the Management of Asthma, updated 2012**. *Thorax* 2008, **63**:Suppl-121.
13. van der Meer V, van Stel HF, Bakker MJ, Roldaan AC, Assendelft WJ, Sterk PJ, RABE KF, Sont JK, SMASHING (Self-Management of Asthma Supported by Hospitals I, Nurses and General practitioners) Study Group: **Weekly self-monitoring and treatment adjustment benefit patients with partly controlled and uncontrolled asthma: an analysis of the SMASHING study**. *Respiratory Research* 2010, **11**:74.
14. Armour C, Bosnic-Anticevich S, Brillant M, Burton D, Emmerton L, Krass I, Saini B, Smith L, Stewart K: **Pharmacy Asthma Care Program (PACP) improves outcomes for patients in the community**. *Thorax* 2007, **62**(6):496-502.
15. Wilson SR, Strub P, Buist AS, Knowles SB, Lavori PW, Lapidus J, Vollmer WM: **Shared treatment decision making improves adherence and outcomes in poorly controlled asthma**. *AmJ RespirCrit Care Med* 2010, **181**(6):566-577.
16. Rabe KF, Adachi M, Lai CKW, Soriano JB, Vermeire PA, Weiss KB, Weiss ST: **Worldwide severity and control of asthma in children and adults: the global asthma insights and reality surveys**. *Journal of Allergy and Clinical Immunology* 2004, **114**(1):40-47.
17. Partridge MR, van der Molen T, Myrseth SE, Busse WW: **Attitudes and actions of asthma patients on regular maintenance therapy: the INSPIRE study**. *BMCPulmMed* 2006, **6**:13.
18. Williams MV, Baker DW, Honig EG, Lee TM, Nowlan A: **Inadequate literacy is a barrier to asthma knowledge and self-care**. *Chest* 1998, **114**(4):1008-1015.
19. Rosenfeld L, Rudd R, Emmons KM, Acevedo-Garcia D, Martin L, Buka S: **Beyond reading alone: the relationship between aural literacy and asthma management**. *PatientEducCouns* 2011, **82**(1):110-116.
20. Hoskins G, McCowan C, Donnan PT, Friend JA, Osman LM, Asthma US, Department SEH: **Results of a national asthma campaign survey of primary care in Scotland**. *International Journal for Quality in Health Care* 2005, **17**(3):209-215.
21. Williams B, Steven K, Sullivan FM: **Tacit and transitionary: An exploration of patients and primary care health professionals goals in relation to asthma**. *Social Science & Medicine* 2011, **72**(8):1359-1366.
22. Clark NM, Nothwehr F: **Self-management of asthma by adult patients**. *PatientEducCouns* 1997, **32**(1 Suppl):S5-20.
