# Supplementary material for: Details of development of the resource for adults with asthma in the RAISIN (randomized trial of an asthma internet self-management intervention) study
Source: BMC Med Inform Decis Mak. 2015 Jul 28;15:57. doi: 10.1186/s12911-015-0177-z (PMC4517557; doi:10.1186/s12911-015-0177-z)
Supplement: Additional file 2: — Additional sample screenshots from Living Well with Asthma. [file 12911_2015_177_MOESM2_ESM.pdf]

Living well with asthma

Thank you for logging in!

The aim with asthma is to have no symptoms and no lifestyle limitations. While this is not possible ALL of the the time, it should be the case MOST of the time. To help with this we would like to send you the occasional email (once every 2 months). If you want to find out more, or prefer not to receive any emails click here.

To help you get information that is right for you it is good to find out a little more about you and your asthma.

Tick any that apply:

	YES
Does your asthma ever stop you doing things you would like to do? (exercising, working, gardening, housework, visiting friends for example)	☐
Does it sometimes affect your sleep?	☐
Do coughs and colds sometimes cause your asthma to flare up?	☐
Do you often have to use your blue/reliever inhaler more than twice a week?	☐
Have you had an asthma attack (e.g needing steroid tablets) in the last 6 months?	☐
Do you currently smoke (even if only occasionally)?	☐

back next

Living well with asthma

What's next?

Next, we need to know if you use a preventer inhaler or not. Telling us this will help give you the right advice.

Preventer inhalers contain steroids (and sometimes other medicines as well) and need to be taken regularly, not just when you have symptoms.

Choose the option which matches your situation best.

- ○ I have never been prescribed a preventer inhaler.
- ○ I have been prescribed a preventer inhaler but don't really use it.
- ○ I mostly/always use my preventer inhaler as prescribed.

back next

Living well with asthma

Why does it matter if people put up with symptoms?

The last page showed that people often don't realise they could feel better if they were on the right treatments.

When people who need a preventer inhaler take it regularly they:

- Can get on with life more normally
- Have fewer asthma attacks
- End up in hospital less

Preventer inhalers contain steroids, and sometimes other medicines too (called combination inhalers).

"Certainly since I've been taking the preventer more seriously, twice a day like I'm supposed to, I've hardly had symptoms, very few. I know that hopefully if I have one of my reactions it won't be as severe as it could be."

back

next

Living well with asthma

My asthma and treatments | Asthma review and action plans | Common concerns and queries | Exercise, stress and anxiety | Take the 4 week challenge | Like to stop smoking? | Useful links and info

Why should I increase my activity levels?

If you were able to watch the video you will know that ANY physical activity is good for your health.

Basically anything that makes you breathe a little faster and feel warmer is helpful.

This doesn't have to be about joining a gym, or playing a sport. Little things in everyday life can add up to make a real difference to your health and wellbeing.

A big review of 19 different studies showed that exercise in asthma is safe, increases fitness levels and improves health related quality of life!

back

next
